# Neural anti-inflammatory action mediated by two types of acetylcholine receptors in the small intestine

**DOI:** 10.1038/s41598-019-41698-w

**Published:** 2019-04-10

**Authors:** Hitomi Kimura, Yu-ki Imura, Hirotaka Tomiyasu, Taiki Mihara, Noriyuki Kaji, Koichi Ohno, Toshihiro Unno, Yasuyuki Tanahashi, Tong-Rong Jan, Hirokazu Tsubone, Hiroshi Ozaki, Masatoshi Hori

**Affiliations:** 1 Department of Veterinary Pharmacology, Graduate School of Agricultural and Life Sciences, The University of Tokyo, Bunkyo-ku, Tokyo 113-8657 Japan; 2 Department of Veterinary Internal Medicine, Graduate School of Agricultural and Life Sciences, The University of Tokyo, Bunkyo-ku, Tokyo 113-8657 Japan; 3 Laboratory of Pharmacology, Department of Veterinary Medicine, Faculty of Applied Biological Science, Gifu University, Gifu, 501-1193 Japan; 4 Department of Animal Medical Sciences, Faculty of Life Sciences, Kyoto Sangyo University, Motoyama, Kamigamo, Kita-Ku, Kyoto 603-8555 Japan; 5 Department and Graduate Institute of Veterinary Medicine, School of Veterinary Medicine, National Taiwan University, Taipei, 10617 Taiwan; 6 Research Center for Food Safety, Graduate School of Agricultural and Life Sciences, The University of Tokyo, Bunkyo-ku, Tokyo 113-8657 Japan

## Abstract

Gastrointestinal prokinetic agents function as serotonin-4 receptor (5-HT_4_R) agonists to activate myenteric plexus neurons to release acetylcholine (ACh), which then induce anti-inflammatory action. Details of this pathway, however, remain unknown. The aim of this study is to clarify the anti-inflammatory mechanism underlying the 5-HT_4_R agonist, mosapride citrate (MOS)-induced anti-inflammatory action on postoperative ileus (POI). POI models were generated from wild-type C57BL6/J (WT), 5-HT_4_R knock-out (S4R KO), α7 nicotinic AChR KO (α7 R KO), and M2 muscarinic ACh receptor KO (M2R KO) mice. MOS attenuated leukocyte infiltration in WT. MOS-induced anti-inflammatory action was completely abolished in both S4R KO and S4R KO mice upon wild-type bone marrow transplantation. MOS-induced anti-inflammatory action against macrophage infiltration, but not neutrophil infiltration, was attenuated in α7 R KO mice. Selective α7nAChR agonists (PNU-282987 and AR-R17779) also inhibited only macrophage infiltration in POI. MOS-mediated inhibition of neutrophil infiltration was diminished by atropine, M2AChR antagonist, methoctramine, and in M2R KO mice. Stimulation with 5-HT_4_R inhibits leukocyte infiltration in POI, possibly through myenteric plexus activation. Released ACh inhibited macrophage and neutrophil infiltration likely by activation of α7nAChR on macrophages and M2AChR. Thus, macrophage and neutrophil recruitment into inflamed sites is regulated by different types of AChR in the small intestine.

## Introduction

Postoperative ileus is the intestinal dysmotility induced by mechanical stimulation of the intestine during laparotomy. The main symptoms of postoperative ileus are abdominal pain, vomiting, halted defecation, and abdominal distension. Postoperative ileus generally improves by itself, but the symptoms sometimes continue. Postoperative ileus can lead not only to paralytic and adhesive ileus, but also to sepsis. Therefore, postoperative ileus is considered as a major problems in the context of gastrointestinal disease^1^. In the early period of postoperative ileus, lingering anaesthetic effects and sympathetic nerve tone suppress gastrointestinal motility. Later, local gastrointestinal inflammation suppresses gastrointestinal motility, and this inflammation is believed to aggravate postoperative ileus.

The currently known pathogenic mechanism of postoperative ileus is briefly outlined below^2–5^. Initially, intestinal manipulation during abdominal surgery activates macrophages in the intestine. Then, these cells produce inflammatory cytokines and chemokines, which induce infiltration of monocyte-derived macrophages and neutrophils that infiltrate the intestinal muscularis. Next, these inflammatory cells produce prostaglandin E_2_ (PGE_2_) via cyclooxygenase (COX)-2 induction and nitric oxide via inducible nitric oxide synthase (iNOS) induction. Finally, PGE_2_ and nitric oxide decrease the contraction of gastrointestinal smooth muscle^6^.

Gastroprokinetic agents are one of the major therapeutic medications for postoperative ileus because they promote the prevention of intraperitoneal adhesion^1^. The serotonin (5-hydroxytriptamine; 5-HT) 4 receptor (5-HT_4_R) agonist mosapride citrate stimulates cholinergic motor neurons in the myenteric plexus of the gastrointestinal tract to release acetylcholine (ACh), which in turn induces gastroprokinetic action^7^. Indeed, results of a clinical trial demonstrate that mosapride citrate significantly ameliorated POI, resulting in reduced hospitalization^8^. Recently, we made the novel finding that mosapride citrate ameliorated gastric ulcer and postoperative ileus through anti-inflammatory action^9,10^. We speculated that stimulation of the 5-HT_4_R of the myenteric plexus in the gastrointestinal tract leads to the release of ACh from the myenteric plexus, and this stimulates α7nAChR on macrophages^10^. Stimulation of α7nAChR has anti-inflammatory effects in many types of inflammatory conditions^11^. For example, a vagovagal cholinergic anti-inflammatory reflex via the afferent vagus nerve/spinal cord/splenic plexus/spleen pathway has been suggested^12^. α7nAChR stimulation is involved in the vagovagal anti-inflammatory reflex. Vagal nerve stimulation activates α7nAChRs that are expressed in splenic macrophages that migrate into inflamed intestinal regions; and these effects mediate the anti-inflammatory effects of vagal stimulation in mice with postoperative ileus^3,13^. It has also been reported that efferent vagal nerve stimulation attenuates gut barrier injury^14^. Detailed anti-inflammatory pathways, however, remain to be elucidated following stimulation of the 5-HT_4_R of the myenteric plexus in the gastrointestinal tract. Therefore, the aim of this study is to clarify the anti-inflammatory mechanism of myenteric plexus stimulation via 5-HT_4_R on leukocyte infiltration in postoperative ileus by using 5-HT_4_R knock-out mice (S4R KO mice), α7nAChR knock-out mice (α7 R KO), and muscarinic 2 AChR knock-out mice (M2R KO mice).

Our results indicate that stimulation of the myenteric plexus via 5-HT_4_R induces two distinct anti-inflammatory signalling pathways; where one inhibits macrophage infiltration via α7nAChR on macrophages, and the other inhibits neutrophil infiltration via M2AChR.

## Results

### 5-HT_4_R stimulation inhibits ileal inflammation induced by intestinal manipulation

**Figure 1:**
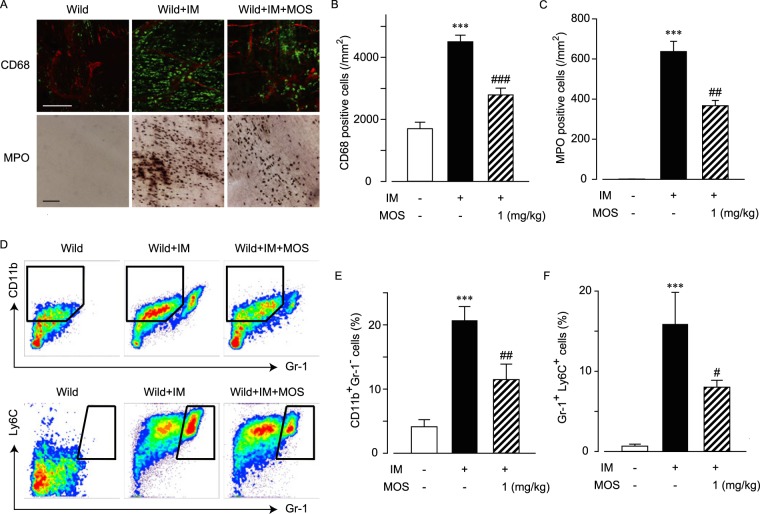
5-HT_4_R stimulation inhibited leukocyte infiltration in postoperative ileus models of WT mice. Effects of 5-HT_4_R stimulation on leukocyte infiltration by intestinal manipulation (IM) in postoperative ileus models of WT mice. Myenteric nerve plexus stimulation via 5-HT_4_R was performed by administration of mosapride citrate (MOS; 1 mg/kg, s.c.) as described in the Methods. (**A**) Immunohistochemical or histochemical staining of CD68-positive macrophages or MPO-stained neutrophils in WT mice intestine. Representative images from 4 independent experiments are shown. Bar indicates 100 μm. Red and green signals indicate PGP 9.5-positive myenteric neurons and CD68-positive macrophages, respectively. (**B** and **C**) Quantified results of infiltrated macrophage (**B**) and neutrophil (**C**) cell numbers from A, where *** indicates values significantly different from control at *p* < 0.001, and ^##^ or ^###^ indicates values significantly different from IM at *p* < 0.01 or 0.001, respectively (n = 4 each). Each column shows the mean ± SEM. (**D**) Typical results of flow cytometry to detect CD11b^+^Gr-1^−^ macrophage subsets (upper trace) or Gr-1^+^Ly6C^+^ neutrophil subsets in POI models of WT mice. Representative results of flow cytometry from 4 independent experiments are shown. (**E** and **F**) Quantified results of % of CD11b^+^Gr-1^−^ macrophage subsets (**E**) and Gr-1^+^Ly6C^+^ neutrophil subsets (**F**) of total leukocytes, where *** indicates values significantly different from control at *p* < 0.001 and ^#^ or ^##^ indicates values significantly different from IM at *p* < 0.05 or 0.01, respectively (n = 4 each). Each column shows the mean ± SEM.

We previously reported details of the anti-inflammatory action of mosapride citrate in a rat model of postoperative ileus^10^. Here, we investigated this action in postoperative ileus model of mice. We immunohistochemically observed CD68-positive macrophages (Fig. 1A,B). Some resident macrophages were detected in the myenteric plexus regions of ileums of WT mice. Numerous macrophages infiltrated into the muscle layer 24 h after intestinal manipulation. As we previously reported in rats, stimulation of 5-HT_4_R in the myenteric plexus strongly inhibited macrophage infiltration in WT mice.

We further investigated MPO-positive neutrophil infiltration (Fig. 1A). Almost no neutrophils were found in the myenteric plexus region of control mice; however, numerous neutrophils infiltrated this region after intestinal manipulation, and stimulation of 5-HT_4_R attenuated this infiltration in WT mice (Fig. 1A and C).

Next, we used flow cytometry to improve the quantitative performance for inflammatory cells. In CD45^+^ 7-AAD^−^ live leukocytes, CD11b^+^ Gr-1^−^ macrophage subsets and Gr-1^+^ Ly6C^+^ neutrophil subsets were quantified (Fig. 1D). In WT mice, CD11b^+^ Gr-1^−^ cells increased following intestinal manipulation, and stimulation of 5-HT_4_R by mosapride citrate attenuated this infiltration (Fig. 1D,E). Gr-1^+^ Ly6C^+^ cell numbers also increased following intestinal manipulation, and stimulation of 5-HT_4_R by mosapride citrate significantly decreased these numbers (Fig. 1D and F).

### 5-HT_4_R stimulation-induced anti-inflammatory action is mediated by 5-HT_4_R

It is established that the main metabolite of MOS (M1) exhibits a partial antagonistic action against 5-HT_3A_R. Recent studies revealed that 5-HT_3A_R antagonists also exhibit anti-inflammatory action in gastrointestinal diseases^15^. Given this, we clarified if MOS-induced anti-inflammatory actions are actually mediated through activation of 5-HT_4_R by using S4R KO mice for our experimental model.

**Figure 2:**
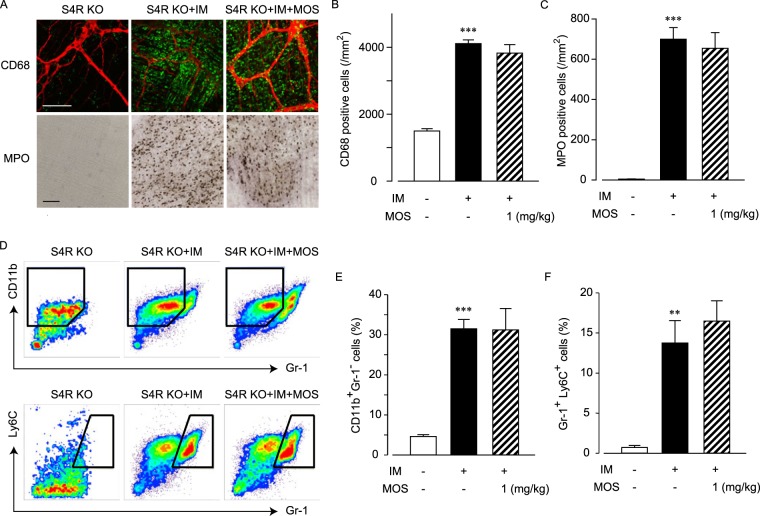
Mosapride citrate-mediated anti-inflammatory action was induced by activation of 5-HT_4_R. Effects of 5-HT_4_R stimulation on leukocyte infiltration by intestinal manipulation (IM) in postoperative ileus model of S4R KO mice. (**A**) Immunohistochemical or histochemical staining of CD68-positive macrophages or MPO-stained neutrophils in S4R KO mice intestine. Representative pictures from 4 independent experiments are shown. Bar indicates 100 μm. Immunohistochemical conditions are the same for Fig. 1A. (**B** and **C**) Quantified results of infiltrated macrophage (**B**) and neutrophil (**C**) cell numbers from A, where *** indicates values significantly different from control at *p* < 0.001 (n = 4 each). Each column shows the mean ± SEM. D: Typical results of flow cytometry to detect CD11b^+^Gr-1^−^ macrophage subsets (upper trace) or Gr-1^+^Ly6C^+^ neutrophil subsets in postoperative ileus models of S4R KO mice. Representative results of flow cytometry from 4 independent experiments are shown. (**E** and **F**) Quantified results of % of CD11b^+^Gr-1^−^ macrophage subsets (**E**) and Gr-1^+^Ly6C^+^ neutrophil subsets (**F**) of total leukocytes, where ** or *** indicates values significantly different from control at *p* < 0.01 or 0.001, respectively. Each column shows the mean ± SEM.

Upon morphological analysis, it was observed that CD68 positive macrophages and MPO positive neutrophils were increased by intestinal manipulation (Fig. 2A). Stimulation of 5-HT_4_R by mosapride citrate did not suppress the macrophage and neutrophil infiltrations in S4R KO mice (Fig. 2A–C). According to flow cytometric analysis, CD11b^+^ Gr-1^−^ macrophages and Gr-1^+^ Ly6C^+^ neutrophils infiltrated following intestinal manipulation (Fig. 2D). Stimulation of 5-HT_4_R did not affect the ratio of both cells in S4R KO mice (Fig. 2D–F). These results demonstrate that the mosapride citrate-induced anti-inflammatory action is mediated through 5-HT_4_R stimulation, and not through 5-HT_3A_R antagonistic action via its metabolite M1.

Additionally, we further investigated the effect of mosapride citrate-induced anti-inflammatory action against macrophage infiltration in POI using S4R KO mice transplanted with bone marrow derived from wild type mice (Supplemental Fig. S1). Results indicated that the mosapride citrate-induced anti-inflammatory action was also abolished in the bone marrow transplanted S4R KO mice possessing wild type bone marrow derived cells.

### 5-HT_4_R stimulation inhibits macrophage infiltration through α7nAChR function and neutrophil infiltration independent of α7nAChR

**Figure 3:**
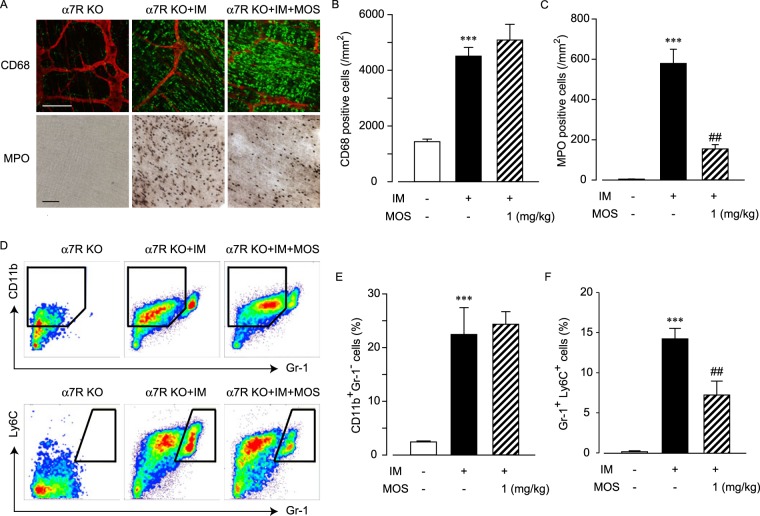
Inhibitory action of 5-HT_4_R stimulation against macrophage infiltration, but not neutrophil infiltration, was attenuated in postoperative ileus models of α7 R KO mice. Effects of 5-HT_4_R stimulation on leukocyte infiltration by intestinal manipulation (IM) in postoperative ileus models of α7 R KO mice. (**A**) Immunohistochemical or histochemical staining of CD68-positive macrophages or MPO-stained neutrophils in α7 R KO mice intestines. Representative images from 4 independent experiments are shown. Bar indicates 100 μm. Immunohistochemical conditions were identical for Fig. 1A. (**B** and **C**) Quantified results of infiltrated macrophage (**B**) and neutrophil (**C**) cell numbers from A, where *** indicates values significantly different from control at *p* < 0.001 (n = 4 each), and ^##^ indicates values significantly different from IM at *p* < 0.01, respectively (n = 4 each). Each column shows the mean ± SEM. (**D**) Typical results of flow cytometry to detect CD11b^+^Gr-1^−^ macrophage subsets (upper trace) or Gr-1^+^Ly6C^+^ neutrophil subsets in POI models of α7 R KO mice. Representative results of flow cytometry from 4 independent experiments are shown. (**E** and **F**) Quantified results of % of CD11b^+^Gr-1^−^ macrophage subsets (**E**) and Gr-1^+^Ly6C^+^ neutrophil subsets (**F**) of total leukocytes, where *** indicates values significantly different from control at *p* < 0.001 (n = 4 each), and ^##^ indicates values significantly different from IM at *p* < 0.01, respectively (n = 4 each). Each column shows the mean ± SEM.

We next investigated the role of α7nAChR in the anti-inflammatory action induced by 5-HT_4_R stimulation. In α7R KO mice, CD68 positive macrophages infiltrated into the inflamed muscle layer as a result of intestinal manipulation, and 5-HT_4_R stimulation by MOS did not attenuate this process (Fig. 3A,B). MPO-positive neutrophils also infiltrated inflamed muscle tissue following IM. Surprisingly, 5-HT_4_R stimulation by mosapride citrate suppressed the neutrophil infiltration, but not macrophage infiltration, even in α7R KO mice (Fig. 3A and C).

Using flow cytometric analysis, similar results were obtained. Specifically, myenteric plexus stimulation via 5-HT_4_R did not attenuate CD11b^+^ Gr-1^−^ macrophages (Fig. 3D,E), but this process did attenuate Gr-1^+^ Ly6C^+^ neutrophils (Fig. 3D and F) after intestinal manipulation in α7R KO mice.

### α7nAChR selective agonists inhibited only macrophage infiltration and not neutrophil infiltration in POI

**Figure 4:**
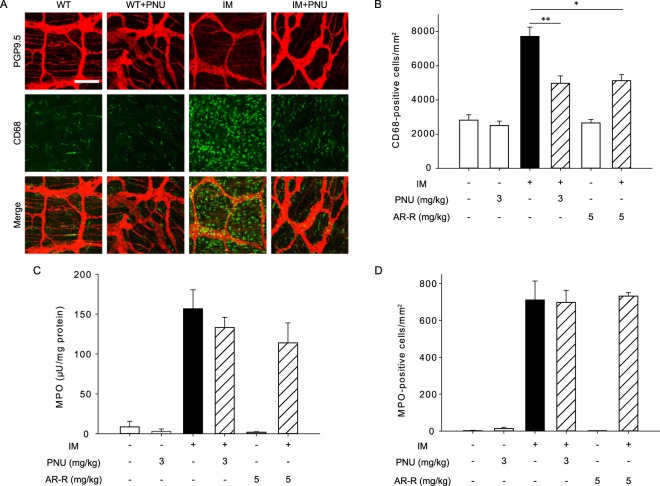
The α7nAChR selective agonists PNU and AR-R inhibited macrophage infiltration, but not neutrophil infiltration, in postoperative ileus models of WT mice. Effects of α7nAChR stimulation on leukocyte infiltration by intestinal manipulation (IM) in postoperative ileus models of WT mice. PNU- (PNU) or AR-R- (AR-R) were subcutaneously administered as described in Methods. (**A**) Immunohistochemical staining of CD68-positive macrophages in postoperative ileus of WT mice. Representative images from 4 independent experiments are shown. Bar indicates 100 μm. (**B**) Quantified results of infiltrated macrophage cell numbers from A, where * or *** indicates values significantly different from control at *p* < 0.05 or 0.001 (n = 4 each), respectively. Each column shows the mean ± SEM. (**C**) Effect of PNU or AR-R on increased MPO activity of inflamed intestinal muscle layer in postoperative ileus of WT mice. Bars show the means ± SEM (n = 4–6). (**D**) Quantitative results of infiltrated neutrophil cell numbers from histochemistry results. Each column shows the mean ± SEM (n = 4).

We further investigated the effects of the selective α7nAChR agonists PNU-282987 (PNU) and AR-R17779 (AR-R) on leukocyte infiltration in postoperative ileus. In WT mice, PNU and AR-R exhibited no effects on leukocyte infiltration (Fig. 4). Administration of PNU or AR-R significantly inhibited macrophage infiltration, but not neutrophil infiltration, following intestinal manipulation in WT mice (Fig. 4A,B and D). Additionally, PNU and AR-R did not inhibit MPO activity resulting from intestinal manipulation (Fig. 4C). We further confirmed the specific neutrophil subset by using Ly6G (1A8-Ly6G; 127607, BioLegend) instead of Gr-1 (RB6-8C5; 12-5931; eBioscience) when evaluating the anti-inflammatory effects of PNU. As expected, PNU also did not attenuate neutrophil infiltration, as indicated by the presence of Ly6G^+^ Ly6C^+^ cells (Supplemental Fig. S2).

**Figure 5:**
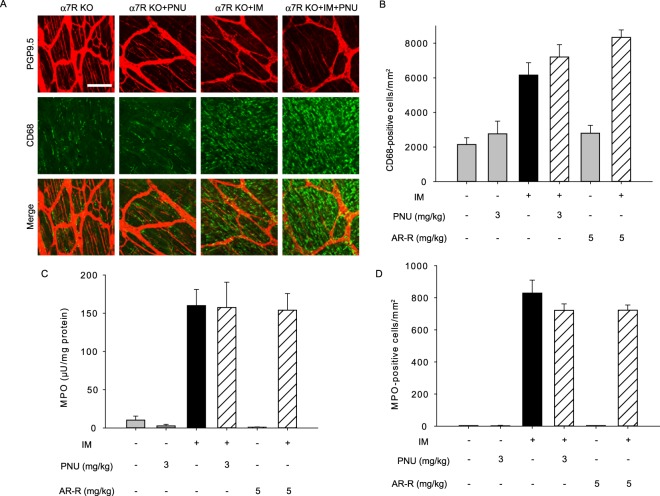
Inhibitory action of the α7nAChR agonists PNU and AR-R on macrophage infiltration was attenuated in postoperative ileus models of α7 R KO mice. (**A**) Immunohistochemical staining of CD68-positive macrophages in postoperative ileus of α7 R KO mice. Representative images from 4 independent experiments are shown. Bar indicates 100 μm. (**B**) Quantified results of infiltrated macrophage cell numbers from A. Each column shows the mean ± SEM (n = 4). (**C**) Effect of PNU or AR-R on increased MPO activity of inflamed intestinal muscle layer in postoperative ileus of α7 R KO mice. Bars show the means ± SEM (n = 4–6). (**D**) Quantitative results of infiltrated neutrophil cell numbers from histochemistry results. Each column shows the mean ± SEM (n = 4).

We next examined the effects of PNU and AR-R on leukocyte infiltration following intestinal manipulation in α7 R KO mice (Fig. 5). Results indicated that the inhibitory action of both compounds against macrophage infiltration was completely abolished in α7 R KO mice. Taken together, we concluded that the anti-inflammatory activity induced by myenteric plexus stimulation via 5-HT_4_R in the context of macrophage invasion is mediated through α7nAChR, but the inhibitory action against neutrophil infiltration is independent of α7nAChR.

Finally, we confirmed α7nAChR expression in the inflamed myenteric plexus region using post-operative ileus models (Supplemental Fig. S3). These results indicated that α7nAChR is expressed in CD68-stained macrophages within the inflamed muscle layer.

### 5-HT_4_R stimulation inhibits neutrophil infiltration via M2AChR

**Figure 6:**
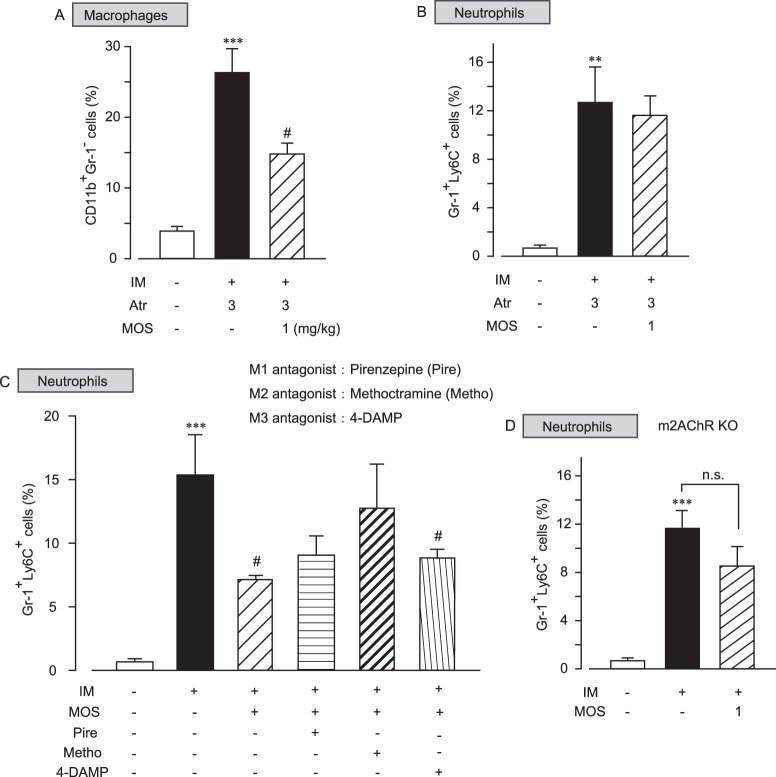
Inhibition of M2AChR prevented the anti-inflammatory action of 5-HT_4_R stimulation against neutrophil infiltration, but not macrophage infiltration, in POI models. (**A** and **B**) Effect of the nonselective mAChRs antagonist atropine on mosapride citrate (MOS)-induced anti-inflammatory action against CD11b^+^Gr-1^−^ macrophage infiltration (**A**) and Gr-1^+^Ly6C^+^ neutrophil infiltration. Atropine (Atr; 3 mg/kg) was subcutaneously administered 10 min before each application of MOS, and *** indicates values significantly different from control at p < 0.001, and ^#^ indicates values significantly different from IM at p < 0.05 (n = 4 each). Each column shows the mean ± SEM. (**C**) Effects of specific mAChR antagonists on the MOS-induced anti-inflammatory action against Gr-1^+^Ly6C^+^ neutrophil infiltration in postoperative ileus. The M1AChR antagonist pirenzepine (Pire; 1 mg.kg), the M2AChR antagonist methoctramine (1 mg/kg), and the m3AChR antagonist 4-DAMP (1 mg/kg) were subcutaneously administered 10 min before each application of MOS, and *** indicates values significantly different from control at p < 0.001, and ^#^ indicates values significantly different from IM at p < 0.05 (n = 4 each). (**D**) MOS-induced anti-inflammatory action against Gr-1^+^Ly6C^+^ neutrophil infiltration in postoperative ileus models of M2AChR KO (M2R KO) mice, where *** indicates values significantly different from control at p < 0.001, and n.s. indicates non-significant values.

5-HT_4_R stimulation activates cholinergic neurons of the myenteric plexus to release ACh. Released ACh can activate α7nAChR, which in turn inhibits macrophage infiltration in postoperative ileus. Given this, we hypothesized that inhibitory action against neutrophil infiltration caused by 5-HT_4_R stimulation may be mediated through muscarinic AChRs (mAChRs). We next investigated the effect of atropine on inhibitory action against neutrophil infiltration caused by 5-HT_4_R stimulation (Fig. 6A).

CD11b^+^ Gr-1^−^ macrophage infiltration was induced by intestinal manipulation. Macrophage infiltration was attenuated by mosapride citrate treatment even in the presence of atropine (Fig. 6A). Conversely, the mosapride citrate-mediated anti-inflammatory action targeting Gr1^+^ Ly6C^+^ neutrophil infiltration was abolished in the presence of atropine (Fig. 6B), suggesting that 5-HT_4_R stimulation inhibits neutrophil infiltration via mAChRs. To determine the subtype of mAChRs that contribute to 5-HT_4_R-mediated anti-inflammatory action against neutrophils, the M1, M2, or M3 selective antagonists pirenzepine, methoctramine, or 4-DAMP, respectively, were employed. In the presence of methoctramine, but not M1 or M3 antagonists, 5-HT_4_-mediated anti-inflammatory action against neutrophil infiltration was reduced (Fig. 6C). To confirm this, we examined the effects of 5-HT_4_R stimulation by mosapride citrate against neutrophil infiltration in M2R KO mice (Fig. 6D). Intestinal manipulation increased the number of Gr1^+^ Ly6C^+^ neutrophils observed in M2R KO mice in a manner similar to that in WT mice. Mosapride citrate-induced inhibitory action against neutrophil infiltration was weakened in M2R KO mice (Fig. 6E). These results indicated that anti-inflammatory action induced by 5-HT_4_R stimulation against neutrophil infiltration is predominantly mediated through M2AChR activation.

## Discussion

Many CD68-positive cells (monocytes and macrophages) and MPO-positive cells (neutrophils) infiltrated the ileal muscularis 24 h after intestinal manipulation. In particular, in addition to dendritic resident macrophages, numerous round, CD68-positive macrophages derived from monocytes infiltrated the ileal muscularis. Thus, intestinal manipulation activated resident macrophages in the intestinal myenteric plexus region, resulting in local inflammation of leukocytes into the muscle layer as reported previously^2,3,10^. Flow cytometric analysis also demonstrated this inflammatory event in postoperative ileus. Populations of CD11b^+^-Gr-1^−^ macrophage subsets and Gr-1^+^-Ly6C^+^ neutrophil subsets were increased following intestinal manipulation.

Administration with 5-HT_4_R agonists significantly reduced the number of infiltrated CD68-positive macrophages and MPO-stained neutrophils in the intestine of mice with postoperative ileus, and this was in agreement with previous results observed in rat models^10^. The anti-inflammatory action of the 5-HT_4_R agonist mosapride citrate was abolished by autonomic ganglionic blockers^10^. As 5-HT-mediated interneurons can activate cholinergic motor neurons in myenteric plexus, it is likely that stimulation of 5-HT_4_R within the gastrointestine activates the myenteric plexus to release ACh. Flow cytometric analysis revealed that populations of CD11b^+^-Gr-1^−^ macrophage subsets and Gr-1^+^-Ly6C^+^ neutrophil subsets found in postoperative ileus were also decreased by 5-HT_4_R stimulation. Taken together, our results indicate that stimulation of the myenteric nerve plexus via 5-HT_4_R inhibits leukocyte infiltration induced by intestinal manipulation in the ileum of postoperative ileus mouse models. Additionally, this mosapride citrate-induced anti-inflammatory action was not observed in post-operative ileus models of chimeric S4R KO mice that received wild type bone marrow transplants, suggesting that mosapride citrated-induced anti-inflammatory action is not mediated by bone marrow-derived immune reactive cells such as macrophages.

It was previously reported that the main metabolite of mosapride citrate M1 exerts an antagonistic action on 5-HT_3A_R^16^. Recently, 5-HT_3A_R antagonists also induced anti-inflammatory action in gastrointestinal diseases^15,17^. Given this, the possibility that the anti-inflammatory action of mosapride citrate might be mediated through its main metabolite, M1, must be investigated. To address this issue, we examined the anti-inflammatory action of mosapride citrate against postoperative ileus in S4R KO mice. Our results demonstrated that the anti-inflammatory action mediated by stimulation of the myenteric plexus via 5-HT_4_R was completely abrogated, indicating 5-HT_4_R, but not 5-HT_3A_R, plays a more significant role in mosapride citrate-induced anti-inflammatory action in postoperative ileus.

Stimulation of the afferent vagus nerve by inflammation can activate the hypothalamic- pituitary-adrenal (HPA) axis in the central nervous system, and the HPA axis in turn releases glucocorticoids from the adrenal gland to reduce inflammation. Additionally, a ‘vagovagal cholinergic anti-inflammatory reflex’ via the afferent vagus nerve/spinal cord/splenic plexus/spleen has been suggested^12^. This anti-inflammatory reflex is exerted by activating α7nAChRs of macrophages via T-cell activation in the spleen^18^. In postoperative ileus model mice, vagus nerve stimulation exerts an anti-inflammatory effect^3,13^; however myenteric nerve stimulation via 5-HT_4_R also induced anti-inflammatory action via α7nAChR, as evidenced by results obtained from pharmacological approaches^10^. Given this, we investigated the effects of myenteric nerve plexus stimulation via 5-HT_4_R on leukocyte infiltration caused by intestinal manipulation in postoperative ileus models of α7 R KO mice. Inhibitory action of mosapride citrate against macrophage infiltration was disrupted, while neutrophil infiltration was still inhibited by mosapride citrate in α7 R KO mice. We further investigated the effects of the α7nAChR selective agonists PNU and AR-R on leukocyte infiltration induced by intestinal manipulation in WT mice, and these results indicated that PNU and AR-R inhibited macrophage infiltration, and not that of neutrophils, caused by IM,. These findings differed from those of a previous report^3^. Both α7nAChR agonists exhibited no effect on the increased MPO activity induced by intestinal manipulation. This ameliorative effect of α7nAChR agonists against macrophage infiltration induced by intestinal manipulation was not observed in α7 R KO mice. The traditional Japanese herbal medicine, Daikenchuto, which has a 5-HT_4_R agonistic action similar to mosapride citrate, also inhibited infiltration of macrophages and neutrophils induced by intestinal manipulation^19^. The anti-inflammatory action of Daikenchuto against infiltration of macrophages, however, was reduced in α7 R KO mice in a manner consistent with the current results^20,21^. This reduction was not observed in neutrophils derived from this model. Taken together, these data indicate that myenteric plexus nerve stimulation via 5-HT_4_R in the myenteric plexus region inhibited infiltration of macrophages, but not neutrophils, through α7AChR activation in postoperative ileus. The reason for the discrepancy between our current results and previous reports is currently unclear^3,13^. Tracey *et al*. suggest that the cholinergic anti-inflammatory pathway is mediated by splenic macrophages expressing α7nAChR^12^. Conversely, Coimbra *et al*. found that efferent vagal nerve stimulation directly attenuates local intestinal inflammation^14^. Here, we demonstrated that intestinal macrophages after IM expressed α7nAChR (Supplemental Fig. 3), suggesting that the local pathway from 5-HT_4_R to α7nAChR was involved in the 5-HT4R-mediated anti-inflammatory action observed in the intestine. We cannot, however, rule out the possibility that systemic α7nAChR expressed in macrophages is important for MOS-mediated anti-inflammatory pathway function. Regardless, neuronal anti-inflammatory signalling through myenteric plexus nerve stimulation via 5-HT_4_R in gastrointestine is different from that occurring through vagovagal stimulation. The relationship between 5-HT_4_R stimulation-induced anti-inflammation and α7nAChR-mediated anti-inflammation requires more detailed future study.

We further tried to determine the mechanisms underlying anti-inflammatory signalling by myenteric plexus stimulation via 5-HT_4_R against neutrophil infiltration. As stimulation of 5-HT_4_R in gastrointestine activates the myenteric plexus nerve to release ACh, we hypothesized that activation of mAChRs may be related to inhibition of neutrophil infiltration induced by 5-HT_4_R agonists. Of note, the nonselective mAChR antagonist atropine blocked the inhibitory action against neutrophil infiltration, but not that of macrophages, mediated by myenteric nerve plexus stimulation via 5-HT_4_R. Additionally, pharmacological studies involving the selective M2AChR antagonist methoctramine attenuated anti-inflammatory action against neutrophil infiltration by myenteric nerve plexus stimulation via 5-HT_4_R. These results were supported by our results observed in M2R KO mice. Taken together, the inhibitory action of myenteric nerve plexus stimulation against neutrophil infiltration is mediated, at least in part, through M2AChR stimulation.

We previously reported that muscularis resident macrophages did not bind to α-bungarotoxin in the ileal muscle layer of healthy rats^10^. In contrast, in inflamed muscle layers of the postoperative ileus of rats, numerous α-bungarotoxin-bound cells were stained with the macrophage marker antibodies ED1 and ED2, indicating that activated macrophages may induce α7nAChR. Conversely, a recent report indicated that in mice resident muscularis macrophages can bind α-bungarotoxin, indicating that α7nAChR may be expressed in non-activated muscularis resident macrophages^13^. Further studies are required to identify the subset of macrophages that express α7nAChRs in the intestinal muscle layer of both control and postoperative ileus mouse models.

In our current study, we could not determine the target cells expressing M2AChR. Kawashima and Fujii reported that M4 and M5AChRs were expressed on all mononuclear leukocytes (MNLs), whereas M1, M2, and M3AChRs were variously expressed^22^. Recent work reported that activation of M4AChR, which is classified as the same group with M2AChR for coupling to Gi/o, induced anti-inflammatory action in carrageenan induced paw oedema^23^. Activation of M4AChR may suppress the JAK2/STAT3 signalling pathway and exert anti-inflammatory effects similar to those observed after α7nAChR stimulation. Another report demonstrated that M3AChR stimulation can ameliorate lipopolysaccharide-induced lung inflammation in lung alveolar macrophages in mice^24^. Further study is necessary to ascertain target cells expressing M2AChR in the gastrointestine to inhibit neutrophil infiltration caused by inflammation.

In conclusion, 5-HT_4_R stimulation inhibits leukocyte infiltration in POI, possibly through myenteric plexus activation. Released ACh inhibited macrophage and neutrophil infiltrations, presumably through activation of α7nAChR on macrophages and M2AChR on neutrophils, respectively. Thus, neural anti-inflammatory pathways mediated by gastrointestinal myenteric nerve plexus stimulation by 5-HT_4_R are regulated by two types of acetylcholine receptors.

## Methods

### Animal model of POI

Postoperative ileus models were created by surgical intestinal manipulation of the distal ileali in C57BL/6 J (wild type; WT), S4R KO, α7 R KO, and M2R KO mice. Mice were cared for in strict compliance with the Guide to Animal Use and Care published by the University of Tokyo. The Institutional Review Board of the Graduate School of Agriculture and Life Sciences of the University of Tokyo approved the study protocol. All mice were anaesthetized using sodium pentobarbital at 40 mg/kg i.p. (Somnopentyl; Kyoritsu Seiyaku Corp., Tokyo, Japan), and the animal model of postoperative ileus was made by intestinal manipulation previously reported^4^. Briefly, the distal ileum (10 cm from the ileocecal region) was exposed and scratched three times with a sterile moist cotton applicator. In the present study, laparotomy with intestinal manipulation treatments was considered as a postoperative ileus model.

Five-week-old male S4R KO mice received 9 Gy irradiation for bone marrow ablation. Then, 2 × 10^6^ bone marrow cells obtained from C57BL/6 J donor mice were reconstituted. The mice were used for the experiments at 3 weeks after the transplantation.

### Experimental design

The mice were randomly assigned to the following groups in WT, S4R KO and α7 KO mice. WT, S4R or α7 R KO received no treatment with fasting, + IM (intestinal manipulation); administration of sterilized physiological saline subcutaneously at 2 h before and 2 and 6 h after intestinal manipulation, IM + MOS (mosapride citrate), and the 5-HT_4_R agonist mosapride citrate (1 mg/kg, donated by Sumitomo Dainippon Pharma) was similarly injected three times. Mosapride citrate was dissolved in 1% lactic acid with sterilized physiological saline.

Additional experiments examined the anti-inflammatory effects of the α7nAChR agonists PNU-282987 (PNU; Sigma-Aldrich Japan, Tokyo, Japan) and AR-R17779 (AR-R; Tocris Bioscience, Bristol, UK) in POI. PNU (3 mg/kg), was subcutaneously injected 0.5 h before and 2 h after intestinal manipulation. AR-R (5 mg/kg) was subcutaneously injected 0.5 h before IM. PNU was dissolved in physiological saline, and AR-R was dissolved in 1% dimethylsulfoxide in physiological saline. Each control animal was treated with each solvent.

The Institutional Review Board of the Graduate School of Agriculture and Life Sciences of the University of Tokyo approved the study protocol (permission number, P16-187). All animal care and experiments complied with the Guide for Animal Use and Care published by the University of Tokyo.

### Whole mount immunohistochemistry

Primary and secondary antibodies are listed in Table 1. Mice were exsanguinated and manipulated ileal parts were isolated at 24 h after intestinal manipulation. The ileum was opened along the mesenteric attachment, and the mucosal and submucosal layers were removed with incisive scissors and tweezers. The ileal smooth muscle layer was cut into 0.7 × 0.7 cm pieces and fixed in 5% neutral formalin in Tris-buffered saline (TBS) overnight at 4 °C. The preparations were washed with TBS three times. After membrane permeabilization with 0.5% Triton in TBS for 2 h, primary antibody treatments were performed overnight at 4 °C. Secondary antibodies were then used after washing three times. Then preparations were washed three times with TBS and immunohistochemically analysed using an LSM510 confocal microscope (Carl Zeiss Japan, Tokyo, Japan) and Digital Eclipse C1 (Nikon, Tokyo, Japan). CD68 positive cells in the myenteric plexus layer of four randomly selected areas in each preparation were counted and the average values were calculated. Experiments were performed at least four times to calculate means ± SEM.

**Table 1:** Antibodies used for immunohistochemistry.

Antibody	Dilution
Rabbit anti-human PGP9.5 polyclonal antibody (UltraClone Limited)	1:100
Rat anti-mouse CD68 monoclonal antibody (AbD Serotec)	1:500
Alexa Fluor 488 Donkey Anti-Rat IgG (Invitrogen)	1:500
Alexa Fluor 568 Goat Anti-Rabbit IgG (Invitrogen)	1:500

### Myeloperoxidase staining

Whole mount fixed preparations were washed three times with TBS for 1.5 h. They were incubated in TBS containing 0.1% (w/v) Hanker-Yates reagent (Polysciences, Warrington, Pennsylvania, USA) and 0.03 (v/v) hydrogen peroxidase (Mitsubishi Gas Chemical Company, Tokyo, Japan) for 5 min and then washed in TBS for at least 10 min. Myeloperoxidase (MPO)-positive neutrophils were counted under a microscope (Nikon ACT-1C for DXM1200; Nikon, Tokyo, Japan) in four randomly selected areas of each preparation.

### Flow cytometry

The isolated ileal muscle layer was starved for 30 min in Ca^2+^-free Hank’s solution and then digested for 1 h with continuous stirring at 37 °C in the presence of collagenase (Worthington Type II, Wako), bovine serum albumin (BSA; Sigma-Aldrich Japan), trypsin inhibitor (Sigma-Aldrich Japan), and adenosine triphosphate (ATP; Sigma-Aldrich Japan) in Ca^2+^-free Hank’s solution. Leukocytes were stained with monoclonal antibodies including anti-CD45 (30-F11; 25–0451; eBioecience, San Diego, CA), 7-Aminoactinomysin (7-AAD; 559925; BD Pharmingen Japan, Tokyo, Japan), CD11b (M1/70; 11–0112; eBioscience), Gr-1 (RB6–8C5; 12–5931; eBioscience), and Ly6C (AL-21; 560596; BD Pharmingen Japan). Samples were acquired and evaluated using a FACSVerse (BD Biosciences, Tokyo, Japan). For all samples, approximately 30,000 live 7-AAD-negative cells were analysed for plot generation.

### Statistics

Results are expressed as means ± SEM. Data were statistically evaluated using unpaired Student t tests for comparisons between two groups and by one-way analysis of variance (ANOVA) followed by Dunnett’s test for comparisons among more than two groups. Values of p < 0.05 were considered statistically significant.

## Supplementary information

Supplementary material
