# Supplementary material for: Neural anti-inflammatory action mediated by two types of acetylcholine receptors in the small intestine
Source: Sci Rep. 2019 Apr 10;9:5887. doi: 10.1038/s41598-019-41698-w (PMC6458176; doi:10.1038/s41598-019-41698-w)
Supplement: Supplementary file 1 — Supplementary material [file 41598_2019_41698_MOESM1_ESM.docx]

Supplementary material

**Neural anti-inflammatory action mediated by two types of**

**acetylcholine receptors in the small intestine**

Hitomi Kimura^1)#^, Yu-ki Imura^1)#^, Hirotaka Tomiyasu^2)^, Taiki Mihara^1)^, Noriyuki Kaji^1)^, Koichi Ohno^2)^, Toshihiro Unno^3)^, Yasuyuki Tanahashi^4)^, Tong-Rong Jan^5)^, Hirokazu Tsubone^6)^,

Hiroshi Ozaki^1)^ and Masatoshi Hori^1)*^

^1)^ Department of Veterinary Pharmacology, ^2)^ Department of Veterinary Internal Medicine, Graduate School of Agricultural and Life Sciences, The University of Tokyo, Bunkyo-ku, Tokyo 113-8657, Japan

^3)^ Laboratory of Pharmacology, Department of Veterinary Medicine, Faculty of Applied Biological Science, Gifu University, Gifu 501-1193, Japan

^4)^ Department of Animal Medical Sciences, Faculty of Life Sciences, Kyoto Sangyo University, Motoyama, Kamigamo, Kita-Ku, Kyoto 603-8555, Japan

^5)^ Department and Graduate Institute of Veterinary Medicine, School of Veterinary Medicine, National Taiwan University, Taipei 10617, Taiwan

^6)^ Research Center for Food Safety, Graduate School of Agricultural and Life Sciences, The University of Tokyo, Bunkyo-ku, Tokyo 113-8657, Japan

* Corresponding author

Masatoshi Hori, Ph.D., D.V.M.

Department of Veterinary Pharmacology, Graduate School of Agricultural and Life Sciences, The University of Tokyo. 1-1-1 Yayoi, Bunkyo-ku, Tokyo 113-8657, Japan

Tel: +81-3-5841-7940 E-mail address: ahori@mail.ecc.u-tokyo.ac.jp

#: H.K. and Y.I. contributed equally.

Supplemental Figure Legends.

Figure S1. The effect of MOS in chimeric S4R KO mice which received transplanted wild type bone marrow.

Five weeks old male S4R KO mice received 9 Gy irradiation for bone marrows ablation. Then 2×10^6^ bone marrow cells from C57BL/6 donor mice were reconstituted. The mice were used for the experiments at 3 weeks after the transplantation. POI model was made as described in Methods. Anti-inflammatory effect of mosapride citrate against CD11b^+^Gr-1^-^ macrophage infiltration (A) and Gr-1^+^Ly-6C^+^ neutrophil infiltration (B) in chimeric S4R KO mice.

*** indicates values significantly different from control at p<0.001, and ** indicates values significantly different from control at p<0.01 (Control; n=4, IM and IM+MOS; each n=6). Each column shows the mean ± SEM.

**Figure S2.** **The effect of PNU on macrophage and neutrophil infiltration in post-operative ileus model of WT mice using another set of antibodies.**

Anti-inflammatory effect of α7nAChRs agonist PNU against CD11b^+^ Ly6G^-^ macrophage infiltration (A) and Ly6G^+^Ly6C^+^ neutrophil infiltration (B). PNU was subcutaneously administered as described in Methods.

*** indicates values significantly different from control at p<0.001, and # indicates values significantly different from IM at p<0.05 (n=4 each). Each column shows the mean ± SEM.

**Figure S3. The expressions of α7nAChR on macrophages in POI mice.**

POI model was made and manipulated ileal smooth muscle layers were removed. They were cut into pieces and fixed in acetone and MeOH, each for 10 min at 4°C. The preparations were washed with TBS three times and blocked in 2% BSA in TBS. Primary antibodies were treated overnight at 4°C. Secondary antibodies were treated for an hour at RT after washing three times. Then preparations were washed three times with TBS and immunohistochemically analyzed as described in Methods. Primary and secondary antibodies are listed in Table S1.

# Figure S1


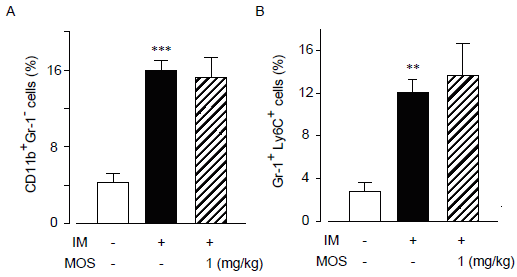


# Figure S2


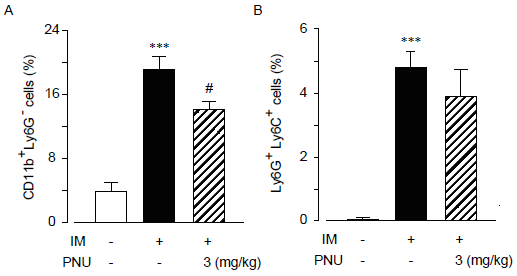


# Figure S3


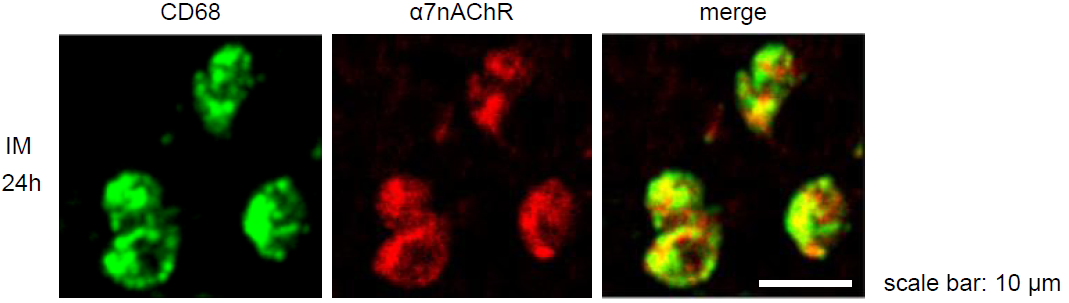


**Table S1** Antibodies used for immunohistochemistry

| Antibody | **Dilution** |
| --- | --- |
| Rat anti-mouse CD68 monoclonal antibody (AbD Serotec) | 1:500 |
| Rabbit anti-α7nAChR polyclonal antibody (Abcam) | 1:100 |
| Alexa Fluor 488 Donkey Anti-Rat IgG (Invitrogen) | 1:500 |
| Alexa Fluor 568 Goat Anti-Rabbit IgG (Invitrogen) | 1:500 |
